# The Discovery of Insulin: An Important Milestone in the History of Medicine

**DOI:** 10.3389/fendo.2018.00613

**Published:** 2018-10-23

**Authors:** Ignazio Vecchio, Cristina Tornali, Nicola Luigi Bragazzi, Mariano Martini

**Affiliations:** ^1^Department of Clinical and Experimental Medicine, University of Catania, Catania, Italy; ^2^Department of Biomedical and Biotechnological Sciences, University of Catania, Catania, Italy; ^3^Department of Health Sciences (DISSAL), Postgraduate School of Public Health, University of Genoa, Genoa, Italy; ^4^Section of History of Medicine and Ethics, Department of Health Sciences (DISSAL), University of Genoa, Genoa, Italy

**Keywords:** discovery of insulin, diabetes, history of medicine, Banting and Best, Paulescu

## Abstract

The discovery of insulin represents an authentic breakthrough, characterized, at the same time, by contrasts, controversies and disputes among scholars, as well as by great disappointments, failures and hopes. It is the story of famous, almost famous and little known people, of serendipities, discoveries and re-discoveries. The discovery of insulin has been a milestone and has truly revolutionized both the therapy and the prognosis of the diabetes, one of the diseases most studied in the history of medicine, whose first mentions trace back to a collection of ancient Egyptian, Indian and Chinese textbooks. As stated by Colwell, the introduction of insulin has heralded the end of the so-called “pre-insulin era” or “frustration era”, paving the way for a new era and clinical advancements. The current review offers a broad, comprehensive overview of main steps culminating into insulin discovery, including recent advancements such as personalized and individualized insulin therapy.

## Introduction

The discovery of insulin represents an authentic breakthrough, characterized, at the same time, by contrasts, controversies and disputes among scholars, as well as by great disappointments, failures and hopes. It is the story of famous, almost famous and little known people (1), of serendipities, discoveries and re-discoveries.

The discovery of insulin has been a milestone in that has truly revolutionized both the therapy and the prognosis of the diabetes. This is one of the most studied diseases in the history of medicine, whose first mentions trace back to a collection of Egyptian medical texts written around 1552 before Christ (BC), the so-called Ebers Papyrus (2–4), and to ancient Indian and Chinese textbooks. The papyrus proposed as a treatment a 4-day course of a decoction of bones, wheat, grain, grit, green lead and earth. The Indian physician, Sushruta, and the surgeon Charaka (400–500 AD) were able to distinguish between a diabetes type 1 and a diabetes type 2, termed as “*madhumeha*” (literally, ‘honey urine’).

The term “diabetes” was probably introduced by the Greek physician Demetrius of Apamea or by Aretaeus of Cappadocia (129-199 AD) from the Greek word “δ*ιαβ*ńτ*ης*” (transliterated “*diab*$\overset{`}{ē}$*tēs*”), literally “passing through” and meaning “siphon” (5). The Roman physician Claudius Galenus (125–199 AD) used the terms “diarrhea urinosa” and “dipsatos,” whereas Avicenna (980–1037 AD), in “The Canon of Medicine,” described abnormal appetite and gangrene in diabetic patients and proposed a mixture of seeds (lupin, fenugreek, and zedoary) as a treatment. The term “diabetes mellitus” was introduced in 1674 by the British physician Thomas Willis (1621–1675) of the Iatrochemical School of medicine (6), for clinically differentiating this disease from the diabetes insipidus, referring to the particular sweetness of urine in diabetic patients (“*quasi melle aut saccharo inbuta*”). Willis defined diabetes mellitus as the “Pissing Evil.” Only in 1776, the Liverpool physician, natural philosopher and experimental physiologist Matthew Dobson (1732 or 1735–1784) (7) discovered that urine of diabetic patients is sweet because of excess in sugar. He published this finding in the “Medical Observations and Enquiries.”

Initially thought to be a kidney disorder, diabetes has been correctly identified as a metabolic pathology. In 1988, during the Banting's lecture, the American endocrinologist Gerald M Reaven, “father of insulin resistance” described the constellation of symptoms now called metabolic syndrome or syndrome X, linking together central obesity (male-type or apple-shaped obesity), diabetes, hypertension, insulin resistance and impaired glucose tolerance.

As stated by Colwell, the introduction of insulin has heralded the end of the so-called “pre-insulin era” or “frustration era,” paving the way for a new era and clinical advancements (8).

## The pre-insulin era

This era is characterized by the efforts of controlling diabetes by means of bizarre pharmacological treatment such as the use of opium or dietary interventions, based on the conviction that diabetic patients should eat extra-portion for compensating their endocrinological and metabolic impairment. In the 1850s the French physician Pierre Adolphe Piorry (1794–1879) prescribed hyper-caloric diets for counteracting the urinary loss of calories (8).

However, some physicians began to notice that it was fasting and not an excess of calories to improve the clinical symptoms of diabetes. In 1706, John Rollo, Surgeon-General to the Royal Artillery, had successfully treated a patient by dietary restriction. The French pharmacist and hygienist Apollinaire Bouchardat (1809–1886), considered the modern father of diabetology, observed an improvement of diabetic patients during the German siege of Paris in 1870. His school, which included the physician Bernhard Naunyin (1839–1925), became famous for advising sugar-free diets, known as the “Bouchardat's treatment” (8). Other nutritional interventions became extremely popular, such as the Allen diet, introduced by the American physician Frederick Madison Allen (1879–1964). It was a carbohydrate-restricted low-calorie diet (9), described in a book entitled “Studies concerning glycosuria and diabetes” and published in 1913. The American physician Elliott Proctor Joslin (1869–1962), founder of the Joslin Diabetes Centre, one of first structures offering specialized service to diabetic patients, was a fervent advocate of a severe, prolonged fasting and of under-nutrition or under-nourishment as a cure for diabetes, the so-called “starvation diet” (9–12).

Insulin would have canceled away all these bizarre treatments, including the oral “miraculous” pills termed metabolin and irrebolin, proposed by Karl Loening in 1922 and by Ernst Vahlen in 1924 (13).

## Toward the discovery of insulin

Insulin is a peptide hormone, which is produced and released by beta cells of the pancreatic islets, that finely tunes the metabolism of carbohydrates, fats and protein inducing the uptake of glucose from the blood into fat, liver and skeletal muscle cells. In 1910 and later in 1916, in London, Sir Edward Albert Sharpey-Schafer (1850–1935) (14) described in depth that the pancreatic islands are able to secrete a substance capable of controlling glucose metabolism, which he termed “insulin,” from Latin “*insula*” (“island”), with reference to the Langherans islands. Other scholars attribute the invention of insulin to the Belgian Jean de Meyer (1878–1934) in 1909.

The history of the steps leading to the discovery of insulin overlaps, at least partially, with the history of diabetes and of pancreatic anatomy. Pancreas (from Greek “$\pi \overset{`}{\alpha }\gamma \kappa \rho \varepsilon \alpha \varsigma$”, literally “all-flesh”) was first identified by the Greek anatomist and surgeon Herophilus (335–280 BC). This term was introduced by the Greek anatomist Rufus of Ephesus (late first century). In 1869, the German pathologist, physiologist and biologist Paul Langerhans (1847–1888) announced that pancreas has two systems of cells (15). In 1875, the German physiologist and histologist Rudolph Heidenhain (1834–1897) performed a study on pancreas and its physiological functions, arriving to state the so-called Heidenhain's law, according to which glandular secretion is always accompanied by modifications and alterations at the level of the anatomic structure of the gland. Further, he had shown that the fresh pancreatic juice was surprisingly not characterized by any proteolytic power (16). This would have been confirmed some years later, in 1902, in France, by the physiologists Camille Délézenne (1868–1932) and Albert Frouin working at the Pasteur Institute. They discovered the enterokinases and characterized the function and activity of the diastases of certain microbes, snakes, plants and poisonous mushrooms (16). However, unfortunately, the discoverers of insulin, Frederick Grant Banting (1891–1941) (17–19) and Charles Herbert Best (1899–1978), ignored this, since they were convinced that pancreatic extract should contain tryptolytic enzymes.

In 1683, in Germany, the Swiss anatomist Johann Conrad Brunner (1653–1727) performed the first pancreatectomy with and without ductal ligation (20). It was however only in 1884 that the French Louis Vaillard (1850–1935) and Charles Louis Xavier Arnozan (1852–1928) discovered that pancreatic duct occlusion caused pancreatic atrophy without hyperglycaemia (8). The closure of the ducts, in fact, led to necrosis of the pancreas, with the exception of the Langerhans islands. This was exploited in 1889 by the German physicians Joseph von Mering (1849–1908) and Oskar Minkowski (1858–1931) (21) who conducted experiments on total pancreatectomy in the dog. Minkowski tried to cure diabetes by injecting pancreatic powder extracts. Further, von Mering was able to induce an experimental diabetes in animals administering phloretin-2′-β-D-glucopyranoside (also known as phloridzin, which actually has been further developed into today's sodium-glucose co-transporter type 2 or SGLT-2 inhibitors such as empagliflozin, canagliflozin, and dapagliflozin) (22). The same experiments were repeated in Italy by Battistini and Capparelli in 1892 and Vanni in 1895 who described results apparently encouraging, but which were clinically non suitable because of the toxicity of pancreatic extracts (23). Capparelli injected triturated fresh pancreas extracts in 0.76% NaCl solution in the abdominal cavity of a dog

In the United Kingdom, Mackenzie and Sibley came to the same results and the same failures.

The Italian endocrinologist and pathologist Giulio Vassale (1862–1913) described (24) that the ligation of Wirsung's duct resulted in atrophy of the esophageal pancreas, sparing the Langerhans islands and, as such, not causing glycosuria. He concluded that the islands had a specific and different function from the rest of the pancreas.

In 1906, the American pathologist and anatomist Lydia Maria Adams DeWitt (23, 24), after ligating the pancreatic ducts of some cats, observed an exocrine pancreas atrophy and, from the Langerhans islands, obtained a beneficial extract for diabetes, which, despite the fact of not being optimal, maintained a discrete glycolytic power. In 1908, the German physician George Ludwig Zuelzer (1870–1949) (23, 24) found favorable results with the administration of pancreatic alcohol extracts in diabetic patients. In 1912, different scholars from many countries including the Italian physicians Massaglia and Zannini (23, 24) came to the conclusion that the destruction of the exocrine pancreas did not produce any glycosuria, which, instead, was manifested after the destruction of the Langerhans Islands.

This discovery led many researchers to use injections of pancreatic extracts to cure diabetes. Studies also aimed at discovering island separation techniques from the rest of the pancreas, with several experiments conducted by Comby in 1892, White in 1893, Johann Karl Goldscheider (1858–1935) in 1894, Wilhelm Sandmeyer in 1895, Doyon in 1897, Hougounena and Doyou in 1897, Hédon in 1898, Blumenthal in 1899, Hess in 1902, Rennie and Fraser in 1907, Raphael Lépine (1840–1919) in 1909, Pratt in 1910, Knowlton and Starling in 1911, the Professor John Murlin and Kramer of the University of Rochester in 1913, Clark in 1916, and Kleiner and Meltzer in 1919, among others (24).

The scientific community has undoubtedly underscored the efforts and merits of the French physiologist and endocrinologist Eugène Gley (1857–1930) (25–27) and of the Rumanian physician Nicolae Constantin Paulescu (1869–1931) (28), who were not sufficiently recognized for their contribution to insulin discovery.

Eugène Gley was inspired by the hypothesis formulated by the French histologist Gustave-Édouard Laguesse (1861–1927) (29, 30) according to which the Langerhans islands secrete a substance capable of preventing the elimination of glucose through the urine. He decided to test this hypothesis with an aqueous extract of pancreas, which was administered to diabetic, pancreatectomized dogs. Gley noted that glycosuria was reduced and the symptoms of diabetes significantly improved.

In order to find out whether, in the animals subject of his experiment, the beneficial action was due to the exocrine pancreas or rather to the action of the islands, he isolated the tissue of the Langerhans islands in the form of an extract to be injected in diabetic dogs. This administration significantly improved and decreased glycosuria. This experiment would have been repeated 25 years later by Banting and Best.

In 1971, Henderson at the question “Who discovered insulin?” replied: “It's about giving Gley the merit of it” (31). After finishing his experiments, Gley wrote a report that sealed within an envelope, which was handed over to the “*Société Francaise de Biologie*” in 1905, with the recommendation to be opened only upon his explicit request. After 1890, Gley no longer repeated his experiments and only in 1921, when Banting and Best made their discovery known in the world, Gley gave the order to open his letter, realizing having discovered the insulin without knowing it!

Another important scholar who should be remembered in the history of insulin discovery is Paulescu, who called it “pancreas”. He studied medicine in Paris where he became assistant to Étienne Lancereaux (1829–1910), who had described in depth the clinical differences between diabetes type 1 and type 2 (“*le diabète maigre et le diabète gras*”) in 1876 (32).

At the age of 31, in 1900, Paulesco returned to Bucharest: despite the young age, he was already considered as an expert in the field of endocrinological studies. In 1904, Paulesco became a professor of physiology at the University of Bucharest until 1931, when he died. As a student, in France, Paulesco was inspired by Mering' and Minkowki's studies, which suggested that the pancreas produced an anti-diabetic hormone.

In 1916, Paulescu, after studying the pancreatectomy in dogs, concluded that the injection of aqueous solution of pancreatic extract allowed an improvement in experimentally induced diabetes.

However, the First World War, already in progress in 1916, blocked Paulescu's studies, which he was able to resume only in 1920 with new experiments whose results were published in 1921 in the journal “*Archives Internationales de Physiologie*.” Paulescu removed the animal pancreas without ligating the excretory ducts, then he emulsified the pancreatic tissue and injected it into the jugular vein of the pancreatectomized dog. In this way, the Romanian physician demonstrated that the extract of the dog and ox pancreas contained some substances capable of acting with an anti-diabetic effect.

However, a dilemma remained unsolved, whether it was possible to separate the substance with anti-diabetic effect produced by the Langherans islands from the rest of the pancreas.

The most used method was always the ligation of the ducts, which would have been later replaced by the use of gelatin for the occlusion of the ducts.

## The insulin era

Banting, a young Canadian orthopedic surgeon, grew up on a small Ontario farm and initially decided to study for ministry at the Victoria College, in Toronto, even though, after some months, he interrupted the study of divinities for the medical studies. During the World War I, he enlisted in the Royal Canadian Army Medical Corps in 1915, and graduated in medicine in December 2016 after attending an accelerated 15-month program. Banting served as a medical officer in England and in France. He was awarded the Military Cross by the British government, for his valorous conduct during the Cambrai campaign (33).

In 1920, Banting, studying the work by Moses Barron (1884–1974) entitled “The relation of the Islets of Langerhans to Diabetes” and published in 1920, was struck by the description of Vaillard' and Arnozan's experiments as well as by the work of Ssobolew, published in this thesis entitled “*Zur normalen und pathologischen Morphologie der inneren Sekretion der Bauchspeicheldrüse*” on the binding of the pancreatic ducts in rabbits, dogs and cats, in order to study the relationship between pancreas and diabetes. Banting was also impressed by the work of E.L. Scott, who, working at the Carlson's laboratory in Chicago, in 1912 came almost to the discovery of insulin, using an alcohol extract, which led him to a step away from the discovery of insulin.

As already mentioned, the difficulty encountered by all scholars was to separate the extract of the Langerhans islands from the rest of the pancreatic exocrine tissue. Banting at the University of Toronto succeeded in this. Since November 1920, Banting began working in a laboratory led by John James Richard MacLeod (33). Banting's goal was to isolate the hormone secreted by the pancreatic islands. Before leaving for a planned holiday in Scotland, MacLeod allowed Banting to be assisted by two young assistants, Best and Noble (34). The researchers closed the pancreatic ducts with a technique designed by Banting to get the degeneration of the pancreatic exocrine tissue and to obtain a pancreatic islet from the pure state. With this liquid extract, for the first time, in the history of medicine, Banting and Best found the way to control glucose in a diabetic animal.

MacLeod, returning from Scotland, guessed the historical importance of the results and, on 11th January 1922, he authorized to conduct experimentation in humans.

Leonard Thompson, a 14-year-old, serious diabetic patient at the Toronto General Hospital, was the first patient to be treated. However, the initial clinical experimentation was a failure: the administration of 15 ml of pancreatic extract had no impact on ketoacidosis, only slightly reducing glycemia and glycosuria, and resulted in the formation of a sterile abscess. On January 23rd Leonard underwent another series of injections and this time he experienced a normalization of glycaemia, glycosuria, and ketonuria. More in details, glycaemia decreased from 520 mg/dl to 120 mg/dl. Glycosuria dropped from 71 to 9 g; ketonuria disappeared. The merit was also of the clinical biochemist James Bertram Collip (1892–1965), who developed a new extraction and concentration protocol. Collip, from the alcoholic acid extract of oxen and pork pancreas, showed that these preparations were more effective than those obtained by the binding of the ducts and subsequent degeneration of the esophagus pancreas.

Besides Leonard, Joe Gilchrist, a diabetic doctor, was another patient who underwent the innovative treatment, and was also the first patient to suffer from hypotensive hyperglycaemia, one of the side-effects of insulin therapy.

On the basis of these successes, on 12th December 1921, Banting and Best reported the results of the discovery of insulin to the American Society of Physiology.

In 1923, a German pharmaceutical laboratory began producing insulin, following the manufacturing license issued by the Toronto Insulin Committee. In 1923, insulin production began in Denmark and Austria, and, in 1924, in Hungary, Australia and Argentina.

In 1923 Banting and MacLeod were awarded the Nobel Prize for Physiology or Medicine. The prize aroused a lively and debated controversy, in that Best, Collip, and Paulescu were excluded. For compensating this, Banting and MacLeod decided to divide their prize with Collip, whilst Noble and Paulescu were officially excluded from the discovery of insulin (34).

## Continuous efforts for improving the quality of insulin

After 1921, scholars intensified their efforts to obtain pure and crystalline insulin preparations. Banting and Best had obtained, indeed short-acting insulin preparations, lasting about 6 h, with inevitable and subsequent peaks of hyperglycaemia and glycosuria, within 24 h. The effort of the experimentations lead to the production of a delayed-acting insulin to counteract both hyperglycaemia and hypoglycaemia.

Hans Christian Hagedorn (1888–1971), who in 1923 formed the Nordisk Insulinlaboratorium, and B.N. Jensen, N.N. Krarup and J. Wodstrup produced in 1936 a slowly absorbed insulin in Copenhagen, combining the hormone with protamine, a basic protein. In 1939, David Aylmen Scott, in Toronto, created the insulin–protamine zinco complex, whose glucose lowering effect lasted up to 48 h.

In 1951–1952, the amorphous “lente” insulins (IZS)—semilente, lente, and ultralente—were developed. In Denmark, Knud Hallas-Møller, the chemical engineers Thorvald (1887–1961) and Harald Pedersen and Jörgen Schlichtkrull, with his method that combined re-crystallization of conventional insulin and chromatographic procedures produced an insulin-slow, protamine-free zinc. Later, the brothers Pedersen decided to leave the Nordisk Insulinlaboratorium and established their own pharmaceutical firm, the Novo Nordisk.

In 1955, the British biochemist Frederick Sanger (1918–2013) managed to fully sequence the bovine insulin and discovered its exact composition in terms of amino-acids. For this discovery, Sanger won the Nobel for Chemistry in 1958. For the discovery of the physical structure of insulin, the English biochemist Dorothy Mary Crowfoot-Hodgkin (1910–1994), a pioneer in the protein X-ray crystallography, was awarded the Nobel Prize in Chemistry in 1964.

In 1956, the American physician and scientist Solomon Berson (1919–1972), with the medical physicist Rosalyn Sussman Yalow (1921–2011), two towering figures in the field of clinical biochemistry, began to develop a radioimmunoassay (RIA) for monitoring glycemia. The assay was successfully completed in 1961. For this, Yalow received the Nobel in Physiology or Medicine in 1977, along with Roger Charles Louis Guillemin (born in 1924) and Andrzej Viktor “Andrew” Schally (born in 1926).

In 1963 and 1965, independently P.G. Seeing in the USA, Wangyu in the Popular Republic of China and H. Zahn, in Western Germany were able to synthesize insulin.

Insulin discovery also aroused other discoveries, such as glucagon, a hormone produced by the alpha cells of the Langerhans Islands, with hyperglycaemic action, opposed to that of insulin, discovered by the British C.W.A. Kimbell. In 1973, Roger Guillemin identified somatostatin, a pituitary polypeptide, capable of reducing the hyperglycaemia in insulin-free diabetes. efforts to improve insulin lead to Allen's myrtillin (an anthocyanin, a 3-glucoside of delphinidin) and Collipìs glucokinin.

Insulin purification techniques were improved until the preparation of insulin with a human-like chemical structure. Subsequently, human insulin was produced using the recombinant DNA technique, thanks to the genetic modification of bacteria.

In 1975, fully synthetic insulin (CGP 12 831) was synthesized in the laboratories of Ciba-Geigy in Basel. Genentech rDNA human insulin obtained on 24th August, 1978 from combination of A and B chains individually expressed in *E. coli*. In 1980, recombinant DNA human insulin was first tested in a sample of 17 non diabetic volunteers, in England. The first diabetic patient treated was Sandy Atherton, 37-year-old, from Wichita, Kansas (USA). In 1982 other synthetic insulins, much more less allergenic than animal insulins, became widely available to diabetic patients, such as Humilin, manufactured by Eli Lilly. On 10th April 1986 the approval to market BHI derived from human proinsulin was signed. In the 1980-90s analog insulins were produced, that is to say a genetically modified form of insulin where the amino acid sequence has been altered in order to optimize insulin absorption, distribution, metabolism and excretion. In 1996, Eli Lilly introduced the first type of analog insulin lispro under the brand name of Humalog. Aspart was approved and released in 2000, while glulisine in 2004.

Concerning needle-free insulins, Exubera, the first inhaled insulin, has been developed by Sanofi-Aventis and Pfizer and marketed by Pzifer in 2006. The idea of administering insulin by breathing an aerosol can be found for the first time in 1925 and has been subsequently relaunched in 1971 (35–37). Investigations carried out by Pfizer Inc. in partnership with Nektar on aerosolized formulations led to the development of a dry powder with appropriate particulate characteristics for deposition in the alveoli. Inhaled insulin has low bioavailability (about 9% of the amount inhaled) but results in adequate serum insulin levels, therefore being as effective as subcutaneously administered insulin at controlling glucose levels in both type 1 and type 2 diabetes. However, in October 2007 Pfizer decided to cease sales of the product. Insulin delivered through the AERx insulin Diabetes Management System AERx iDMS (Aradigm, Hayward, CA), or other delivery systems, such as ProMaxx, AIR, Spiros, and Technosphere/Afrezza (MannKind, Danbury, CT) and Solo products with inhaled insulin (38–49).

Another promising product is the buccal/oral insulin. Oralin was approved in 2005 in Ecuador and marketed by Generex uses RapidMist technology to deliver a mixture of insulin, surfactants and lipids to buccal mucosa (50). Further advances in technology include the Emisphere technologies, such as the Eligen technology, and the Nobex technology, which is able to deliver covalently modified insulin, namely hexyl insulin monoconjugate 2 (HIM2) across membranes (51).

## Toward personalized insulin therapy

After the discovery of insulin and its synthesis, another important step was to make the patient share with the physician the insulin therapy, that is to say to acquire the ability to control one's own blood and urine with glucose dosage, in such a way that the treatment and the management become truly personalized. With this regard, it should be briefly mentioned the development of reactive strips to control glycaemia, glycosuria and acetonuria, which have made diabetic patients protagonists in the daily management of their disease. In 1963 Dr. Arnold Kadish of Los Angeles, California, designed the first insulin pump to be worn, which had more or less the size of a Marine backpack (52). In 1979, Al Mann CEO of PaceSetter Systems became interested in insulin pumps. In 1983, MiniMed introduced the model 502. In1986 MiniMed introduced the so-called “insulin friendly tubing.” In 1992, in 1996 and in 1999, MiniMed launched the models 506, 507C and 508, respectively. In 2001 MiniMed was acquired by Medtronic.

In 1967, Updike and Hicks realized a miniature electrical transducer of glucose and implanted it in an animal in order to monitor glucose continuously (53). In the 1970s, the group of John Pickup and Harry Keen (54, 55) as well as the group of Tamborlane (56) proposed the continuous intra-cutaneous injection of insulin (CSII). In the following years, other groups refined this approach and tried to close the loop integrating the pump and the continuous glucose monitor (CGM): namely, Albisser (57), Pfeiffer (58), Mirouze (59), Kraegen (60) and Shichiri (61), among others. The combination of CSII with CGM devices results into sensor augmented pumps (SAPs). As of today, there are more than 140 trials on closed loop pumps, including intra-peritoneal or dual hormone pumps.

An individualized approach of insulin therapy can improve glycemic control, minimizing hypoglycemic risk and side-effects, conjugating patient preferences and increasing adherence to treatment (62).

## The post-insulin era: beyond insulin?

The dream of Banting was to transplant pancreas in humans (63). The first who attempted to graft pancreatic tissue to cure diabetes was the English doctor P. Watson Williams from Bristol, who, on the 20th December 1893, grafted three fragments of a pancreas obtained from a sheep into the subcutaneous tissue of a 15-year-old boy suffering from diabetes (64, 65). The English surgeon Frederick Charles Pybus (1883–1975), from Newcastle-on-Tyne, reported unsuccessful attempts in 1916–1924 (66, 67). The first successful transplantation of pancreatic tissue was a whole organ pancreas transplant in 1966 carried out by the group of Kelly (65).

Artificial pancreas (AP) is another promising approach to go beyond insulin. Cobelli and coworkers (68) have defined AP as a “closed-loop control of blood glucose in diabetes, is a system combining a glucose sensor, a control algorithm, and an insulin infusion device.” So far, only one type of bedside-type artificial endocrine pancreas is currently available: STG-22 (Nikkiso Co. Ltd., Japan) (69).

In conclusion, the discovery of insulin is not only a milestone in the history of medicine—still ongoing (Table 1)—but also an important lesson, with different, both historical and bioethical implications (70).

**Table 1:** Main events and steps in the history of the discovery of insulin.

**Year**	**Major event**
1552 BC	One of the earliest, if not the earliest, descriptions of diabetes
1675	Thomas Willis coins the expression “diabetes mellitus”
1682–1709	Johann Conrad Brunner observes polydipsia and polyuria in dogs after partial pancreatectomy
1706	John Rollo, Surgeon-General to the Royal Artillery, treats a patient by dietary restriction
1776	Matthew Dobson discovers that urine of diabetic patients is sweet because of excess in sugar
1788	Thomas Crawley reports some clinical cases and links diabetes with pancreatic dysfunctions
1850s	French physician Pierre Adolphe Piorry introduces hypercaloric dietary treatment
1857	French Claude Bernard links diabetes with excess glucose production
1860s	Claude Bernard reports extensive physiopathological mechanisms of diabetes
1865	Introduction of the concept of “opotherapy” by Ancelet
1869	Paul Langerhans discovers cellular islets within the pancreas Noyes reports a case of retinitis in diabetic patients
1870s	Bouchardat postulates a link between pancreas and diabetes
1874	Kussmaul describes the air hunger of ketoacidosis in diabetic patients
1875	Heidenhain demonstrates that extracts of the fresh pancreas possess no proteolytic capacities Nikolaus Friedreich postulates an indirect link between diabetes and pancreatic disorders mediated by sympathtetic nerve ganglia, *in primis* celiac ganglion and celiac plexus
1876	Étienne Lancereaux describes in depth the clinical differences between diabetes type 1 and type 2
1889–1893	German physiologist Oskar Minkowski and physician Joseph von Mering, show that if the pancreas is removed from a dog, the animal gets diabetes
1892	Battistini and Capparelli try to cure diabetes by injecting pancreatic powder extracts
1893	Laguesse terms the pancreatic cellular islets after Langerhans (“islets of Langerhans”) On December 20, 1893, Dr. P. Watson Williams in Bristol, England, performs the first intervention of pancreas transplant
1898	Joslin proposes opium as treatment of diabetes
1901	Eugene L Opie correlates the hyaline degeneration of the islets of Langerhans with the occurrence of diabetes
1906	Wilhelm Heiberg develops a method for counting the islets of Langerhans and shows that they are low in diabetic patients
1907	J Rennie and T Fraser, from Aberdeen Royal Infirmary, experiment the effect of extracts of pancreatic islets from various *teleostei*, in particular the *Lophius piscatorious*, to a sample of 5 diabetic patients
1908	Zuelzer experiments pancreatic extracts termed Acomatol Zuelzer submits an application to the USA office of patents
1909	Forschbach publishes his findings of experiments with the Zuelzer's pancreatic extracts
1909–1910	Introduction of the name “insulin” by Belgian Jean de Meyer
1910	Joseph Pratt published “The relation of the pancreas to diabetes” in JAMA
1912	Zuelzer takes out an American patent entitled “Pancreas Preparation Suitable for the Treatment of Diabetes” (serial number 431,226)
1913	Murlin and Kramer propose that an optimal treatment of diabetic patients should lead to the restoration of respiratory quotient
1914	Rose experiments an alkaline pancreatic extract
1915–1919	Meltzer and Kleiner experiment the effect of a pancreatic solution highly diluted in NaCl
1916	Introduction of the name “insuline” by Sir Edward A Sharpey-Shafer of Edinburgh
1918	Hagedorn and the pharmacist Bierger Norman Jensen publish a micro-method for the determination of blood glucose
1920	Moses Barron publishes his “The relation of the islets of Langerhans to diabetes, with special reference to cases of pancreatic lithiasis”
1921	Dr Frederick Banting and medical student Charles Best perform experiments on the pancreases of dogs in Toronto, Canada Nicolae Paulescu presents his findings at the Rumanian Biological Association and later publish them in the “Reports of the French Society of Biology” and in the “*Archives Internationales de Physiologie*” Paulesco terms the impaired hormone in diabetic patients as pancreatin
1922	Bovin insulin was first given injected to humans Banting and Best publish their article entitled “The Internal Secretion of the Pancreas” in the “Journal of Laboratory and Clinical Medicine”
1923	Commercialization of Iletin Banting and Macleod are awarded the Nobel Prize in Physiology or Medicine August Krogh brought insulin to Scandinavia Petrén publishes his “Diabetes-studier”
1924	First attempt to graft pancreatic tissue to cure diabetes by the English surgeon Frederick Charles Pybus
1925	One of the earliest, if not the earliest, mentions of the possibility of administering insulin by breathing an aerosol
1926	JJ Abel obtains the first crystallization of insulin
1936	Sir Harold Percival (Harry) Himsworth introduces the concept of insulin resistance Protamine was utilized to produce a slow-release insulin: Hans Christian Hagedorn introduced protamine insulin in Denmark
1949	Production of the first standardized insulin syringes
1951	Development of amorphous lente insulins (IZS)
1955	Sanger characterized the amino acid sequence of human insulin
1957	Introduction of the technique of immunoassay by Solomon Berson and Rosalind Yalow
1959	Sanger is awarded the Nobel Prize in Chemistry
1960	First prototype of ambulatory insulin pump by Dr. Arnold Kadish
1963	Introduction of insulin pumps
1964	Dorothy Mary Crowfoot-Hodgkin is awarded the Nobel Prize in Chemistry
1966	First successful transplantation of pancreatic tissue by the group of Kelly
1967	Updike and Hicks realize an implantable miniature electrical transducer of glucose
1970s	Continuous subcutaneous insulin infusion (CSII)
1972	Introduction of the first standardized U100 insulin
1974	Development of monocomponent MC insulin or single peak insulin Gérard Slama and colleagues in Paris show that a few days of open-loop intravenous (IV) insulin infusion in type 1 diabetes using a portable pump held in a shoulder bag produce good glycaemic control
1975	Synthesis of the first fully synthetic insulin CGP 12831 Lowy reports the clinical effectiveness of using SMBG in pregnancy
1978	David Goeddel and colleagues of the biotechnology firm Genentech use recombinant DNA techniques to produce synthetic “human” insulin Development of the first portable insulin pumps, the Autosyringe, also named ‘Big Blue Brick’
1979	Introduction of the first needle-free insulin delivery system by Derata
1980	Introduction of the “basal-bolus” concept and intensive insulin therapy
1982–1983	Commercialization of the first insulins utilizing rDNA technology, Humulin® R (rapid) and N (NPH, intermediate-acting)
1983	Commercialization of the Nordisk infuser
1985	Novo Nordisk introduces the Insulin Pen delivery system
1988	Gerald Reaven identifies and describes the constellation of symptoms now called metabolic syndrome
1992	Medtronic releases the MiniMed 506 insulin pump
1993	The Diabetes Control and Complications Trial shows the linear relation between the degree of glycemic control and complications Commercialization of the first instant glucose tablets
1996	Approval of Lispro
2000	Approval of Aspart
2004	Approval of Glulisine
2005	Approval of Detemir Approval of the buccal insulin, Oralin® Approval of the venom of the lizard *Gila monster*, which contains exendin 4, a molecule fostering one of the insulin-releasing pathways
2006	Approval of Exubera, the first inhaled insulin
2008	Approval of the FreeStyle Navigator CGM from Abbott
2013	Rejection of Degludec The University of Cambridge develops an artificial pancreas that pairs the technology of an insulin pump with a continuous glucose monitor
2015	Approval of Degludec Dr Edward Damiano introduces the iLet

## Author contributions

IV and CT conceived the manuscript. IV, CT, NB, and MM drafted the manuscript.

### Conflict of interest statement

The authors declare that the research was conducted in the absence of any commercial or financial relationships that could be construed as a potential conflict of interest.
